# Editorial: Uncertainty Induced Emotional Disorders During the COVID-19

**DOI:** 10.3389/fpsyg.2022.943966

**Published:** 2022-07-05

**Authors:** Fushun Wang, Fang Pan, Yi-Yuan Tang, Jason H. Huang

**Affiliations:** ^1^Institute of Brain and Psychological Science, Sichuan Normal University, Chengdu, China; ^2^Department of Medical Psychology, Shandong University Medical School, Jinan, China; ^3^Department of Psychological Sciences, Texas Tech University, Lubbock, TX, United States; ^4^Department of Neurosurgery, Baylor Scott & White Health, Temple, TX, United States; ^5^Department of Surgery, Texas A&M University College of Medicine, Temple, TX, United States

**Keywords:** uncertainty, basic emotions, core affect, arousal, emotional disorder, COVID-19 editorial, uncertainty induced emotional disorders

The globally devastating COVID-19 was unexpected for most of us. The unexpected high contagion and death rate have impaired the social lives of people all around the world, making many people panicked, anxious, and stressful (Aqeel et al., 2022). The urgent social restriction and nucleic acid testing added further psychological stress. It is reported that more than 30% of people around the world suffer from mental health problems (Levine et al., 2022). The major reason for these psychological problems is due to the uncertainty about COVID-19, for we don't know the death rate of the disease, nor the possibility of getting infected by the disease. In addition, the long-term social restriction further makes people lack information about the conditions of the disease (Daly and Robinson, 2022). In order to resolve these problems, timely knowledge and information, as well as psychological supports, are needed.

Researchers proposed that uncertainty is an important cognitive mechanism for arousal and emotion, and it's also an important cause of various mental disorders such as anxiety and depression (Berchicci et al., 2015). Normally we are calm, but we are aroused if something unexpected happens. The world is full of stimulations, most of which are expected. However, unexpected thing continually occur, for example, we are not sure whether it will rain tomorrow, and the weather forecasts often report that there is an 80% chance of rain. Behavioral economics proposes that most of our thinking is subjected to uncertainty, and most of our choices are not the result of careful deliberations, but result from poor predictors of future behavior, distorted memory, and are affected by our physiological and emotional states (Gu et al., 2021).

Uncertainty plays a very important role in inducing emotions in everyday life, especially for those with affective disorders, and numerous researchers have investigated the power of uncertainty (Gu et al., 2016). For example, the dimensional theory suggested that the external stimulations (and the emotions they induced) have two properties:1) hedonic value, which represents whether they fit into our physiological needs; 2) safety value, which represents whether they happen as expected (Zheng et al., 2016). The hedonic value depends on whether the stimulation fits into our needs: if yes, we will be happy; if not, we will be sad. The safety value depends on whether the stimulations happen as expected: if yes, we are calm; if the stimulations is un-expected, we will be aroused (Zheng et al., 2016). Hedonic value and arousal are two core effects of human emotions (Hutto et al., 2018), which form the two dimensions for the constructive model of emotions (dimensional theory) (Figure 1).

**Figure 1:**
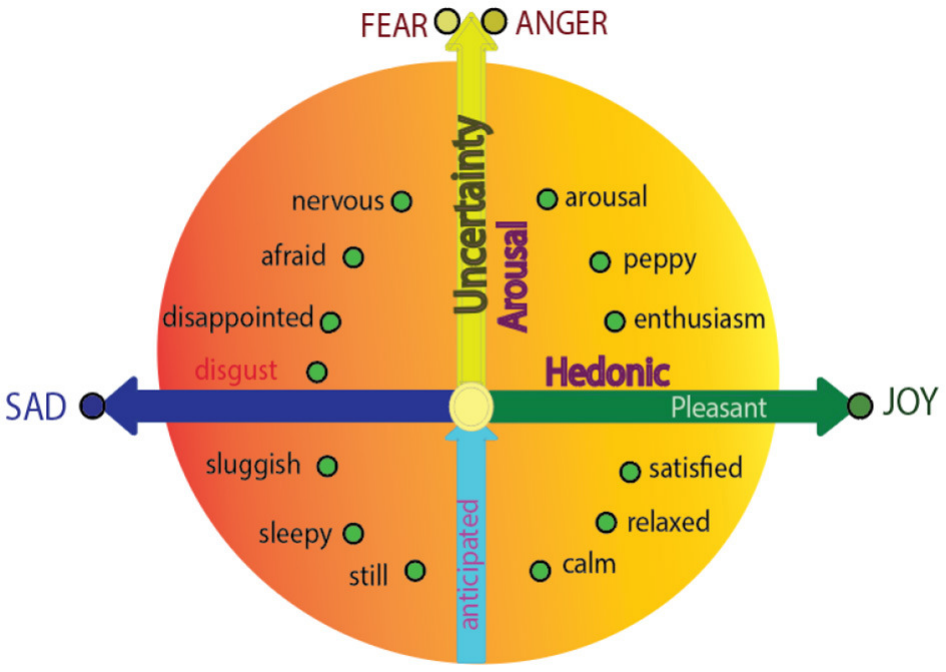
Integrative approach for emotional dimensions and basic emotions. The integrative approach for Basic emotion theory and dimensional theory proposes that the reason that “basic emotions” are “basic” is that the basic emotions are located on the axis of the circumplex (Posner et al., 2009). It means that each “basic emotion” is a special emotion that represents one feature of emotion as a whole, named core affect (arousal or hedonic value) (Wilson-Mendenhall et al., 2013): fear and anger represent the arousal value; while sadness and joy represent the hedonic value, which is related to the hedonic values of the stimulus. Put it another way, “pure” fear and anger are not related to hedonic values; “pure” sad and joy are not related to safety values (modified from previous publications). Please refer to Gu et al. (2019a).

Alternatively, another popular emotional theory proposed that there existed a limited number of emotions, which might include anger, disgust, joy, fear, and sad (Ekman, 1992). However, an integrative approach suggested that the basic emotional theory and dimensional theory are not in conflict with each other, instead, they can be integrated (Panksepp, 2007; Gu et al., 2019a). This integrative theory suggested that basic emotions can also find their locations on the dimensions, like all other emotions. The only specialty for the basic emotions is that they are located on the poles of the dimensions (Figure 1), and they represent different features of emotions. They can also be called core affects, or prototypical emotions. We also suggested that they are subsided by three monoamine neurotransmitters: dopamine-joy, norepinephrine-fear (anger), and 5-HT-disgust (sadness) (Liang et al., 2021). This theory can be called three primary basic emotion theory (Gu et al., 2019b).

Even though there are two major prevalent theories about emotions, both of which agree upon the idea that emotional arousal depends on the un-expectancy of the simulation (Xu et al., 2022). However, it is still not clear how to treat COVID-19-induced psychological problems. So we proposed a topic to collect recent studies about the mental health problems related to the pandemic, including but not limited to review and experimental studies to help people understand COVID-19 induced mental disorders. And in the last year, we have received 39 submissions, and 30 were accepted after peer reviewing.

In the experimental report titled “*Predicting the Severity of Symptoms of the COVID Stress Syndrome From Personality Traits: A Prospective Network Analysis*,” Taylor et al. recruited 1976 participants from US and Canadian adults, and found that intolerance of uncertainty is a personality trait that is related to negative emotion during COVID-19.

In the paper titled “*Mental Health in COVID-19 Pandemic: A Meta-Review of Prevalence Meta-Analyses*,” Sousa Junior et al. presented a meta-review of studies about the mental problems, including depression, anxiety, stress problems, etc, and they found that the rate of mental health problems ranged from 20-36%, which is higher than most expected.

In Musetti et al.'s paper, titled “*Maladaptive Daydreaming in an Adult Italian Population During the COVID-19 Lockdown*,” the authors recruited 6,277 Italian adults and investigated their mental health problems, including negative stress, anxiety and depression, and compulsive fantasy activities, and found that social restriction might exert additional stresses for these problems.

In the paper titled “*The Prevalence of Psychological Status During the COVID-19 Epidemic in China: A Systemic Review and Meta-Analysis*,” Li, Zhang et al. did a meta-analysis of 67 papers, and they found that fear and stress symptoms are common, and anxiety and depression were also prevalent among the public.

In Di Trani et al.'s paper “*From Resilience to Burnout in Healthcare Workers During the COVID-19 Emergency: The Role of the Ability to Tolerate Uncertainty*,” the authors investigated the mental health problems of medical staff in Italy, and the found that tolerance of uncertainty might be the major reason for the mental health problems emergent in COVID-19.

In the paper titled “*A 6-Month Follow-Up Study on Worry and Its impact on Well-Being During the First Wave of COVID-19 Pandemic in an Italian Sample*,” the author Ongaro et al. also investigated the health problems of the Italian people, and they found that mental health policymakers should make some policy to spread the information of the virus contagions, as well as making a longitudinal evaluation about its effects.

In the paper “*Gender Differences in Anxiety, Depression, and Nursing Needs Among Isolated Coronavirus Disease 2019 Patients*,” Li, Li et al. investigated the gender differences in anxiety and depression during COVID-19, and they found that men become more easily worried and stressed at the pandemic compared with their female colleagues.

However, in another study by Song et al., titled “*Psychological Resilience as a Protective Factor for Depression and Anxiety Among the Public During the Outbreak of COVID-19*,” the author did a thorough investigation on the mental status of 3,180 subjects, and they found that women weremore easily stressed, in addition to younger and less educated people.

Similarly, in Nia et al.'s paper, “*Predictors of Persistence of Anxiety, Hyper-Arousal Stress, and Resilience During COVID-19 Epidemic: A National Study in Iran*,” the authors assessed anxiety, hyper-arousal stress, and psychological resilience in the people of Iran, and they also found that young, female, and less educated people experienced more stress.

In Yan et al.'s paper titled “*Mental Health of Pregnant and Postpartum Women During the Coronavirus Disease 2019 Pandemic: A Systematic Review and Meta-Analysis*,” the authors did an investigation on mothers, fetuses, and children, and they found that the prevalence rates were much higher than normal populations.

In the paper titled “*Comorbid Anxiety and Depression and Related Factors Among Pregnant and Postpartum Chinese Women During the COVID-19 Pandemic*,” the authors Luo, Xue et al. also reported that the economically poor, pregnant and postpartum Chinese women more easily encountered mental stresses during COVID-19.

In Li, Liang et al.'s paper, titled “*Social Support, Attachment Closeness, and Self-Esteem Affect Depression in International Students in China*,” the authors investigated the psychological conditions of students from abroad and found that their problems were even worse.

In the paper titled “*Association of Stress-Related Factors With Anxiety Among Chinese Pregnant Participants in an Online Crisis Intervention During COVID-19 Epidemic*,” Shangguan, Wang et al. investigated anxiety problems in pregnant women and suggested it is critically important to continually check on fetal development during COVID-19.

In the paper titled “*Perceived Stress, Resilience, and Anxiety among Pregnant Chinese Women during the COVID-19 Pandemic: Latent Profile Analysis and Mediation Analysis*,” the authors Luo, Shen et al. also tried to introduce some ways to prevent stress for pregnant women during the pandemic and suggested that psychological intervention to reduce stress is a good way to alleviate the psychological problems.

In the paper titled “*Prevalence of Risk Factors Associated With Mental Health Symptoms Among Outpatient Psychiatric Patients and Their Family Members in China During the Coronavirus Disease 2019 Pandemic*,” Qiu et al. investigated the mental health problems of outpatients and found their worries are even more serious, because of economics and nursing burdens.

In the paper “*A Conditional Process Model to Explain Somatization During COVID-19 Epidemic: The Interaction Among Resilience, Perceived Stress and Gender*,” Shanguan, Zhou et al. reported another study to probe into the mechanisms for mental health problems and suggested that psychological resilience is a key predictor of somatization as well as mental problems.

In the paper titled “*Distress, Appraisal, Coping Among the Frontline Healthcare Provider Redeployed to the Epicenter in China During COVID-19 Pandemic*,” the author Ji et al. evaluated the mental health problems of the medical staff on the frontline in treating the COVID-19 patients and suggested that they are incredibly highly stressed and need more social support.

In the paper titled “*Depression, Anxiety, and Suicidal Ideation in Chinese University Students During the COVID-19 Pandemic*,” the author Zhou et al. investigated 11,133 subjects about their anxiety, depression, and suicidal ideation, and found that social support, good education, and being kept well informed are good ways in reducing suicidal ideation.

In the paper titled “*The Stress and Anxiety to Viral Epidemics-6 Items (SAVE-6) Scale: A New Instrument for Assessing the General Population's Anxiety Response to the Viral Epidemic During the COVID-19 Pandemic*,” the author Chung et al. invented a kind of questionnaire, the Stress, and Anxiety to Viral Epidemics-6 items (SAVE-6) scale, and they explored the validity and usefulness of the questionnaire for measuring the general population's anxiety response among 1,009 respondents.

In “*Psychological Impact of COVID-19 on College Students after School Reopening: A Cross-Sectional Study Based on Machine Learning*,” the author Ren et al. did an investigation of mental health problems for students after returning to school after the social restriction and found that their anxiety and depression symptoms are very serious.

The paper “*Protective Predictors Associated With Posttraumatic Stress and Psychological Distress in Chinese Nurses During the Outbreak of COVID-19*” reported Xia et al.'s study about the mental health problems of the nurses, and found that exercise, enough sleep, and low stress can help them maintain their mental health.

In the paper “*Factors That Influence Perceived Organizational Support for Emotional Labor of Chinese Medical Personnel in Hubei*,” Zeng et al. did an investigation about the mental problems of the medical personnel who first went to Wuhan at the beginning of the pandemic.

The paper by Li, Xu et al. titled “*Sense of Coherence and Mental Health in College Students After Returning to School During COVID-19: The Moderating Role of Media Exposure*,” reported that social restriction increased uncertainty and increased anxious and depressive symptoms.

Lu et al. contributed a paper titled “*Effects of Wise Intervention on Perceived Discrimination Among College Students Returning Home From Wuhan During the COVID-19 Outbreak*,” to propose that discrimination against students from Wuhan might induce psychological stresses for the students.

In the paper titled “*COVID-19-related Daily Stress Processes in College-Aged Adults: Examining the Role of Depressive Symptom Severity*,” the authors Greaney et al. from the University of Texas Arlington, reported that daily exposure to stressful information, rumors, or negative news about the virus might exert a negative effect on the population.

In the paper “*The Relationship Between Perceived Stress, State-Trait Anxiety and Sleep Quality Among Graduating Students During COVID-19 Pandemic*,” Liu, Qiao et al. investigated the somatic and mental problems of graduate students and found that uncertainty or perceived stress can work together with poor sleep to induced mental health problems.

In the prospective paper “*Focus on the Mental Health of Pediatric Medical Workers in China After the COVID-19 Epidemic*,” Liu and Wang tried to give a perspective on the ways to improve the health conditions for Chinese pediatric workers, and suggest ways to develop psychological intervention programs that are tailored to them.

In the paper “*Using Mindfulness to Reduce Anxiety and Depression of Patient With Fever Undergoing Screening in an Isolation Ward During COVID-19 Outbreak*,” the authors Liu, Huyang et al. introduced a short time meditation that might be helpful for people who are waiting in line for a nucleic acid test screening.

In the paper, “*Acupuncture combined With emotional Therapy of Chinese Medicine Treatment for Improving Depressive Symptoms in Elderly Patients With Alcohol Dependency During the Epidemic Period of COVID-19*,” Zhao et al. introduced a method of therapy for mental health problems stemming from COVID-19, and suggested that acupuncture combined with Chinese medicine is helpful for patients.

In Lv et al.'s paper titled “*The Effect of Computerized Cognitive Behavioral Therapy on People's Anxiety and Depression During the Six Months of Wuhan's Lockdown of COVID-19 Epidemic*,” the authors used mindfulness to treat the patients during the outbreak of the pandemic in Wuhan and found that mindfulness is really helping the patients, especially for women and students.

Collectively, these studies have investigated thoroughly about the prevalence of mental health problems after COVID-19, the uncertainty mechanism for these disorders, as well as some therapies for these emotional problems. It is believed that uncertainty plays a very important role in people's emotional induction, which affects people's emotional response or arousal by affecting the expected process. Individuals who are intolerant of uncertainty are also more likely to have emotional disturbances such as worry, anxiety, and fear. Regardless of the probability of a negative event occurring, individuals with a high intolerance of uncertainty strongly believe that the uncertainty situation is unacceptable (Dugas et al., 2001). Therefore, in the current COVID-19 pandemic, controlling uncertainty may become a new way of intervention for various mood and anxiety disorders.

## Author Contributions

FW, FP, Y-YT, and JH wrote the paper. All authors agreed upon publishing the manuscript.

## Funding

The paper was supported by a grant from the Foundation of Humanities and Arts from the Ministry of Education in China (19YJAZH083).

## Conflict of Interest

The authors declare that the research was conducted in the absence of any commercial or financial relationships that could be construed as a potential conflict of interest.

## Publisher's Note

All claims expressed in this article are solely those of the authors and do not necessarily represent those of their affiliated organizations, or those of the publisher, the editors and the reviewers. Any product that may be evaluated in this article, or claim that may be made by its manufacturer, is not guaranteed or endorsed by the publisher.
